# Broadband high-resolution molecular spectroscopy with interleaved mid-infrared frequency combs

**DOI:** 10.1038/s41598-020-75704-3

**Published:** 2020-10-29

**Authors:** A. V. Muraviev, D. Konnov, K. L. Vodopyanov

**Affiliations:** CREOL, College of Optics and Photonics, University of Central Florida, Orlando, FL 32816 USA

**Keywords:** Imaging and sensing, Optical spectroscopy

## Abstract

Traditionally, there has been a trade-off in spectroscopic measurements between high resolution, broadband coverage, and acquisition time. Originally envisioned for precision spectroscopy of the hydrogen atom in the ultraviolet, optical frequency combs are now commonly used for probing molecular ro-vibrational transitions throughout broad spectral bands in the mid-infrared providing superior resolution, speed, and the capability of referencing to the primary frequency standards. Here we demonstrate the acquisition of 2.5 million spectral data points over the continuous wavelength range of 3.17–5.13 µm (frequency span 1200 cm^−1^, sampling point spacing 13–21 MHz), via interleaving comb-tooth-resolved spectra acquired with a highly-coherent broadband dual-frequency-comb system based on optical subharmonic generation. With the original comb-line spacing of 115 MHz, overlaying eight spectra with gradually shifted comb lines we fully resolve the amplitude and phase spectra of molecules with narrow Doppler lines, such as carbon disulfide (CS_2_) and its three isotopologues.

## Introduction

Coherent laser beams in the 3 to 20 μm mid-infrared (mid-IR) region provide a unique prospect for sensing molecules through addressing their strongest absorption bands. Thanks to their coherent and broadband nature, optical frequency combs can probe molecular signatures over an extensive (e.g. more than an octave) spectral span simultaneously^1,2^. When one (or several) of the comb teeth is phase locked to a narrow-linewidth reference laser(s), frequency comb spectroscopy can provide the spectral resolution, which is on par with tunable laser spectroscopy—limited only by the absolute comb-tooth linewidth. However, comb spectroscopy has a strong advantage of massive parallelism of data collection and, most importantly, the absolute optical frequency referencing to an accurate external standard (e.g. atomic clock) over the whole spectral span of the comb. Combs with high degree of phase coherence can be used in the dual comb spectroscopy (DCS)—one of the most advanced spectroscopic techniques. In a dual-comb spectrometer, a sensing comb is transmitted through a sample and then multi-heterodyned against a local oscillator (LO) comb which has a repetition rate $f_{r}$ that differs by a small fraction ${\Delta }f_{r}$ from that of the sensing comb. Compared to classical Fourier transform infrared spectroscopy, DCS demonstrates remarkable improvement of spectral resolution, data acquisition speed, and sensitivity, all at the same time^3^.

With a high degree of mutual coherence between the two combs in a DCS system, it is possible to obtain comb-tooth resolved spectra^3–5^. Using DCS in the near-IR (comb span 1.36–1.69 µm), Zolot et al. resolved the phase and amplitude of over 400,000 individual comb modes at a mode spacing of 100 MHz^5^. In the mid-IR, by utilizing frequency combs near 3.3 µm (spectral span 12 cm^−1^) achieved by applying a nonlinear mixing step to near-IR combs produced by electro-optic modulation (EOM) technique, Yan et al. resolved 1,200 comb lines with the line spacing of 300 MHz^6^. Subsequently, two groups demonstrated comb-tooth resolved spectra over a broad span of frequencies: Ycas et al. resolved 270,000 comb lines between 2.6 and 5.2 µm in four overlapping spectral sequences (with mode spacing 200 MHz and a combined frequency span 1800 cm^−1^)^7^, and Muraviev et al. resolved 350,000 comb lines with a finesse of 4,000 within a single frequency comb spanning 3.1–5.5-µm (mode spacing 115 MHz, frequency span 1400 cm^−1^) and also demonstrated simultaneous detection of more than 20 molecular species in a mixture of gases^8^.

Once comb teeth are resolved, the spectral resolution is defined by the comb tooth linewidth, which can be orders of magnitude narrower than the comb-line spacing. Then, high-resolution measurements can be implemented by interleaving spectra taken with discretely stepped—either comb repetition rate *f*_r_ or carrier-envelope offset (CEO) frequency *f*_ceo_^2,9–13^. For example, using a near-IR comb (span of 1.5–1.64 µm) and a mechanical Fourier transform spectrometer with mode-resolving capability, Rutkowski et al. performed measurements, which yielded 2.4 million sampling points with a step of 20 kHz—performed by interleaving spectra with frequency-shifted comb lines^14^. In the mid-IR, Baumann et al. used difference frequency combs near 3.4 µm (spectral span 30 cm^−1^), to attain a high-resolution spectrum of methane, by interleaving spectra acquired by shifting the combs by 25 MHz—one-quarter of the 100-MHz comb-tooth spacing^10^. Using quantum cascade laser (QCL) combs, Villares et al. performed DCS measurements near 7 µm (span 16 cm^−1^) with the sampling point spacing that was improved from the original comb-tooth spacing of 7.5 GHz to 80 MHz by frequency sweeping the combs via QCL current modulation^15^. Similarly, by utilizing dual QCL combs near 8.3 µm (span 55 cm^−1^), Gianella et al. achieved an improvement in the sampling point spacing from 9.8 GHz to 30 MHz by sweeping the frequencies of both the sensing and LO combs via synchronized current modulation^16^. In the two above QCL scenarios the combs were free running with no absolute optical frequency referencing; rather, the frequency scale was calibrated by comparing the spectra with the HITRAN database^17^. Overall, the spectral width of mid-IR measurements with interleaved combs does not exceed 60 cm^−1^ with one exception of a silicon microresonator comb with a span of 3–3.5 µm that was scanned with a step of 80 MHz via tuning both the pump laser frequency and the cavity resonance. However, the comb lines were scanned over 16 GHz—a small portion of the 127-GHz mode spacing^18^.

One of the challenges of DCS is to get high spectral resolution over a broad bandwidth—required, for example, in applications related to multi-species detection in gas mixtures. To achieve high resolution, one needs long mutual coherence time between the two combs^3^. If the combs are not fully locked, one can track the relative phase drifts and correct for these in real time, at the expense of having two stable continuous-wave reference lasers, as has been demonstrated in the near-IR^19–21^, or perform phase correction by a posteriori data processing, as has been shown in the THz and mid-IR ranges^22,23^. However, both of these methods lack the absolute frequency referencing—a setup needs to be calibrated using, for example, a well-known gas absorption feature.

Degenerate (subharmonic) optical parametric oscillators (OPOs) pumped by mode-locked lasers are noteworthy sources of broadband mid-IR frequency combs^24–28^ and are now used in spectroscopic studies^8,29,30^, random number generation^31^, and in coherent Ising machines^32^. Their key benefits are: low (~ 10 mW) oscillation threshold, extremely broad bandwidth, good stability when the cavity is actively locked to resonance, and high conversion efficiency that can exceed 50%^33^. It has been established that a subharmonic OPO is an ideal coherent frequency divider without any excess phase noise, which rigorously both coherently down-converts and augments the spectrum of the pump frequency comb^34–37^.

Here we demonstrate, using a highly-coherent subharmonic DCS system, the acquisition of > 2.5 million spectral data points over the whole (no gaps) spectrum of 3.17–5.13 µm (frequency span 1200 cm^−1^, 36 THz). The sampling density achieved by interleaving comb-line resolved spectra from consecutively shifted combs was sufficient to fully resolve Doppler-broadened absorption bands of several heavy molecules, such as CS_2_ and OCS, with the main effort focused on CS_2_. Its choice was motivated (i) by its importance for atmospheric chemistry^38^, astrobiology^39^, and medical diagnostics^40,41^, and (ii) by the fact that the accurate high-resolution spectroscopic data for CS_2_ in the mid-IR range were not yet available.

## Experiment

### Dual-comb setup

Our dual-comb system used a pair of subharmonic OPOs based on orientation-patterned GaAs (OP-GaAs) crystal as a χ^(2)^ gain medium, pumped by a highly coherent twin Tm-fiber frequency comb system with a central wavelength of 1.93 µm, pulse duration of 90 fs, repetition rate $f_{r}$≈ 115 MHz, and the average power of 300 mW for each laser^8^.

**Figure 1:**
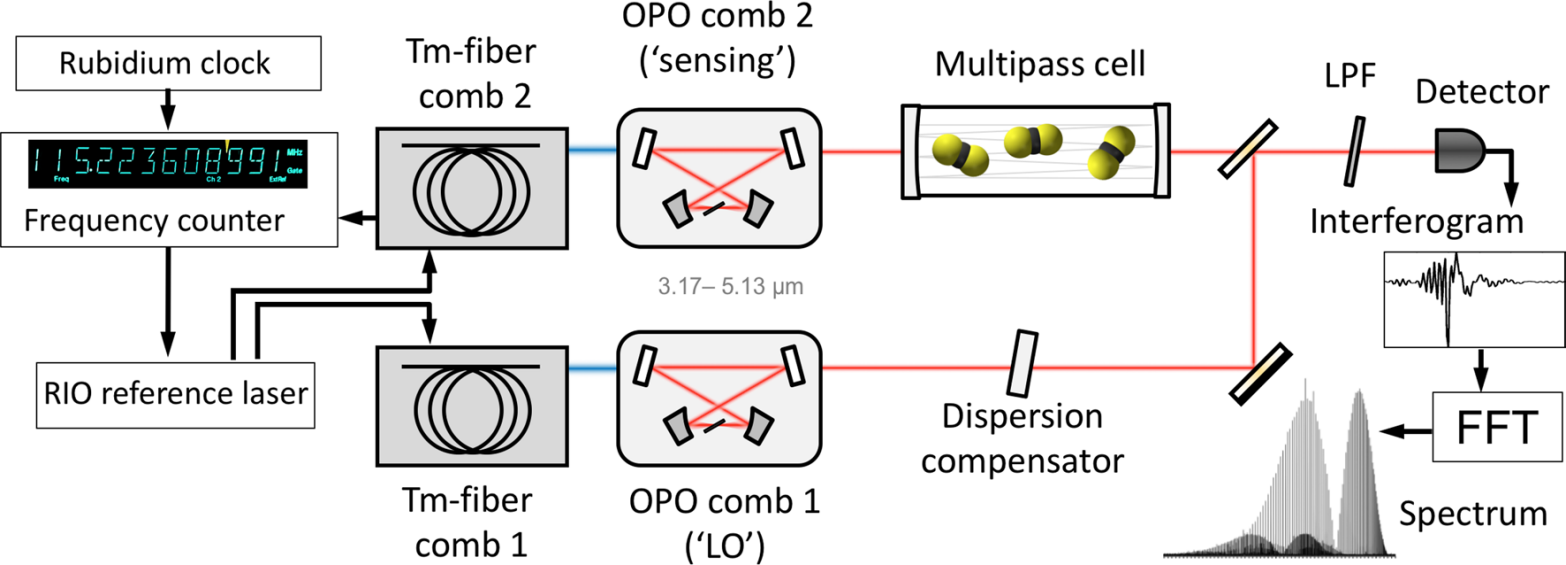
Schematic of the dual-comb spectroscopy setup. LPF, longwave pass filter.

In order to stabilize Tm laser frequency combs, a portion of each laser’s output was used to generate a supercontinuum (SC) in a nonlinear silica fiber. While the 1.1-µm and 2.2-μm SC components were used to stabilize the CEO frequency via *f*–2*f*. interferometry, the component near 1.56 µm was utilized to obtain beat notes with a narrow-linewidth reference diode laser from Redfern Integrated Optics (RIO). Each OPO (Fig. 1) had a ring-cavity bow-tie design with low cavity group delay dispersion (GDD) achieved by (i) using low-dispersion mirrors, (ii) a thin (0.5-mm-long) OP-GaAs crystal, and (iii) an intracavity wedge made of CaF_2_ for GDD compensation^8,36^. The instantaneous spectral coverage of the DCS system was 3.17–5.13 µm (span 1200 cm^−1^) at − 10 dB and 3.08–5.40 µm (span 1400 cm^−1^) at − 20 dB level. The mutual coherence time between the two subharmonic OPOs was previously measured to be as long as 40 s^8^. Our data acquisition electronics and frequency counters were referenced to a Rb atomic clock, which provided ~ 10^−10^ absolute accuracy of the frequency readings.

### Spectroscopic measurements with interleaved combs

We used a one-sided DCS configuration (Fig. 1), where only one (‘sensing’) comb passes through the gas sample. This eliminates the ambiguity of the double-sided scheme, where an absorption profile is probed by both comb lines simultaneously and allows phase spectrum measurement. For absorption measurements we used a multipass gas cell (AMAC-76LW from Aerodyne Research) with 76-m path length and 0.5-L volume. The sensing and LO combs were combined and sent to an infrared detector (InSb from Kolmar, 77 K, 60 MHz) whose output was fed into a 16-bit analogue-to-digital converter (AlazarTech, ATS9626). We used a wedge made of a dielectric (CaF_2_) in the LO channel (Fig. 1) to compensate the dispersion of the multipass cell windows. This did not affect the measured spectrum but made the interferogram sharper, which helped to minimize a jitter in the coherent interferogram averaging process.

First, we demonstrated that an enormous amount of spectral information can be obtained by spectral interleaving over our broadest comb span of 3.17–5.13 µm. The repetition rate offset between the two combs was set to ${\Delta }f_{r}$ = 138.5 Hz, which allowed mapping the whole 1200 cm^−1^-wide optical spectrum into a radiofrequency (RF) range of 1–48 MHz (< $f_{r} /2$, see Methods). For each comb-line-resolved measurement, we coherently averaged data streams consisting of 10 centerbursts spaced by $1/{\Delta }f_{r}$≈7.2 ms, with the coherent averaging time from a few seconds to several hours. For each data stream, the data acquisition process was triggered by the sharp central spike of the interferogram, which provided good repeatability of the waveforms and allowed long (for at least > 10 h) coherent averaging without applying any phase correction procedures. After Fourier transforming the time-domain signal and RF-to-optical frequency up-scaling (see Methods) we obtained a comb-tooth-resolved optical spectrum. By a controlled stepping the optical frequency of the reference RIO laser (see Methods), the comb lines were tuned, thus filling 115-MHz-wide gaps between the comb lines. To fully resolve Doppler-broadened linewidths of species with relatively high molecular weight, such as CS_2_ (Doppler linewidth ~ 90 MHz), we elected to interleave eight comb-line-resolved spectra.

**Figure 2:**
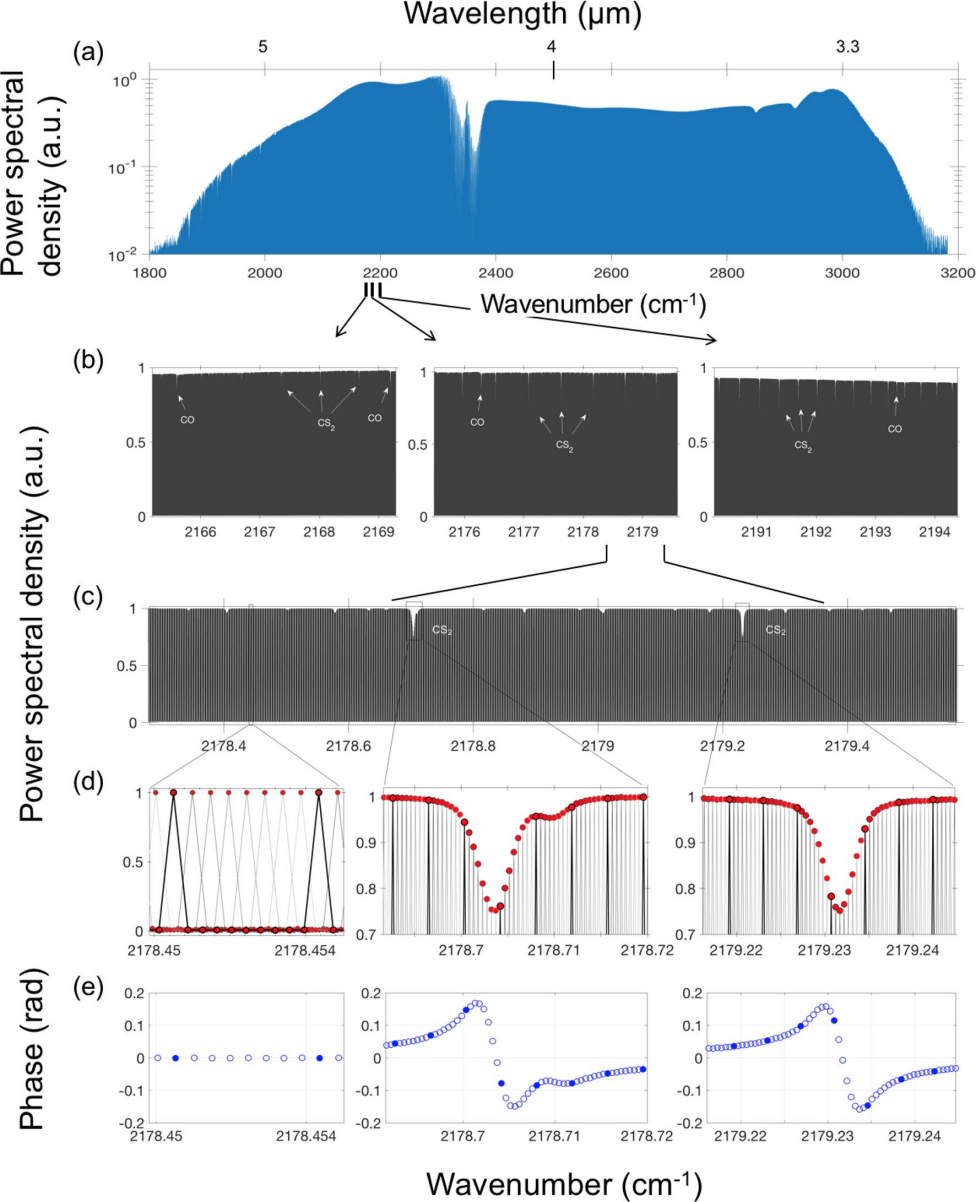
(**a)** Combined mode-resolved spectrum (log-scale) containing > 2.5 million comb lines (− 10-dB level), obtained by eight interleaved measurements. The broad dips in the spectrum are due to the atmospheric absorption (H_2_O, CO_2_) outside the gas cell. (**b**,**c**) Magnified view of the spectrum showing narrow features due to molecular absorption inside the cell. (**d**,**e**) Further magnification shows amplitudes and phases for the individual interleaved comb lines spaced by 14.4 MHz. Features shown in black in (**d**) and filled circles in (**e**) correspond to the original comb-line spacing of 115 MHz.

Figure 2a shows the entire comb-tooth-resolved optical spectrum, consisting of more than 2.5 million comb lines obtained from eight interleaved spectra. The total averaging time was 48 min (6 min for each frequency-shifted measurement). The magnified spectrum, Fig. 2b–d, reveals narrow (Doppler-broadened) absorption dips due to the molecules present in the gas cell (CS_2_, CO), and Fig. 2e shows phases for individual comb lines, modified by molecular resonances.

The comb-line-resolved spectra were obtained in the following way. A digitized (8-ns point spacing, 16-bit vertical resolution) averaged waveform containing *N*_*c*_ centerbursts was resampled to get an integer number of data points *M*_*p*_ (typically expressed as a power of 2) per time interval that is equal to 1/${\Delta }f_{r}$. (In our experiment ${\Delta }f_{r}$ is known with a fractional accuracy of better than 10^–9^, see Methods.) After a Fourier transform, one gets *M*_*p*_* × N*_*c*_ spectral points, where *N*_*c*_-1 points out of *N*_*c*_ are zero (at the noise level), and only one point is non-zero with its value being proportional to the amplitude of a comb line. This may be seen in the zoomed mode-resolved spectrum of Fig. 2d (left panel, black color), which was obtained from a waveform containing *N*_*c*_ = 10 centerbursts. As a result of this procedure, the center frequency for each comb line is well-defined with the fractional uncertainty for the absolute value of ~ $7.8 \times 10^{ - 10}$—through a unique RF-to-optical mapping (see Methods).

## Results

### CS_2_ molecule

**Figure 3:**
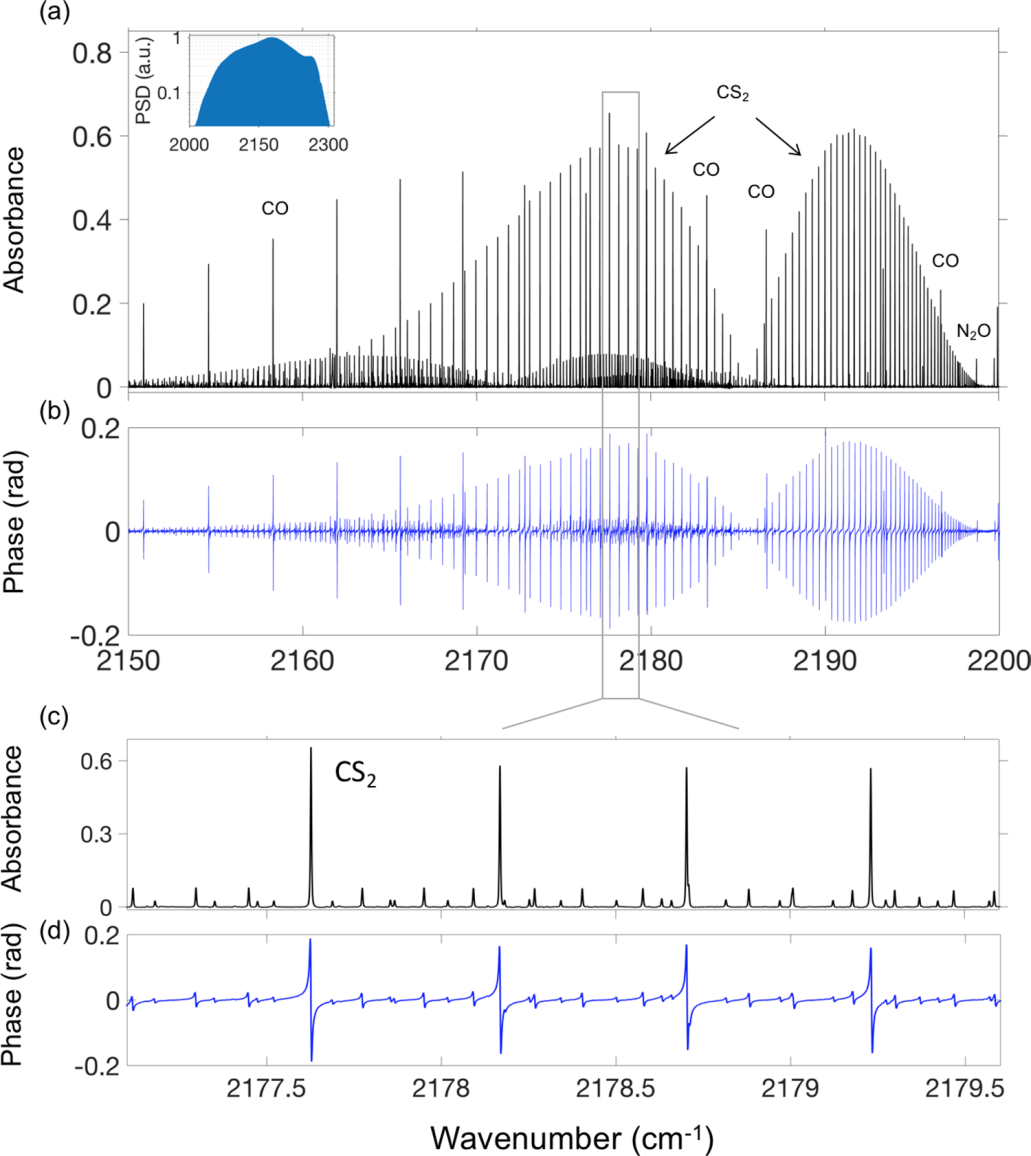
(**a**,**b**) Absorbance ($= - {\ln}\left( {T/T_{0} } \right)$) and phase spectra for the ν_1_ + ν_3_ band of CS_2_ obtained at 14.4-MHz spectral point spacing. (**c**,**d**) Magnified view of the CS_2_ absorbance and phase. The spectral peaks of CO and N_2_O in (**a**,**b**) are due to the room air in the cell. Inset: Reduced comb spectrum (log-scale) used in this experiment. PSD, power spectral density.

For high-precision measurements of the ν_1_ + ν_3_ band of CS_2_, our spectral span was narrowed down to approximately 2030–2300 cm^−1^ (4.3–4.9 µm; – 10-dB level)—achieved by a combination of tweaking the OPO cavities and using a longpass optical filter (the reduced spectrum is shown in the inset to Fig. 3a). The spectral narrowing allowed to increase the signal-to-noise ratio (SNR)^11^ and also increase Δ*f*_r_ by a factor of four, to Δ*f*_r_ = 554 Hz. With the acquisition time of ~ 200 min for each frequency-shifted spectrum, we reached the fractional standard deviation for the comb-line amplitude of $1.5 \times 10^{ - 4}$ (SNR $6.6 \times 10^{3}$). With the average SNR of 4.4 $\times 10^{3}$ over the central (– 4.5-dB level) ≈50,000 modes of the comb (span ≈200 cm^−1^), the DCS figure of merit (*FOM*), identified in^11^ as $SNR \times M/\sqrt \tau$ (*M* is the number of modes and is $\tau$ the averaging time), we get *FOM* = $2.1 \times 10^{6}$ Hz^1/2^.

The gas mixture in the multipass cell had a total pressure of 6.03 mbar and contained a trace amount of CS_2_ at a concentration of 94.6 ± 3 ppm (part-per-million by volume), with n-hexane and room air as buffer gases in similar proportions (see Methods). Figure 3a,b show the absorbance ($A = - {\ln}\left( {T/T_{0} } \right)$, where *T* is the power transmission with, and *T*_0_—without a sample) and phase spectrum corresponding to the ν_1_ + ν_3_ absorption band of CS_2_. A magnified view of the CS_2_ absorbance and phase is given in Figs. 3c,d. The baseline for each interleaved spectrum was obtained by the asymmetric least squares smoothing algorithm based on the Whittaker smoother^42^ with the smoothing parameter that was optimized in such a way that the deviation from the actual data (sufficiently away from a line center) did not exceed the local noise level. Due to the low pressure and high resolution, the spectral lines of other molecules—H_2_O, CO_2_, CO, and N_2_O—present in the gas mixture due to the room air, practically didn’t interfere with the CS_2_ lines. On the other hand, n-hexane, used as a solvent for CS_2_, does not have any characteristic absorption features across the spectral span used here, and gave a very smooth absorption offset. The spectra were obtained from eight interleaved comb-line resolved measurements. Every data point represents a comb line with a known absolute frequency (see Methods). The distance between the sampling points is 14.4 MHz—about 1/8 of the original comb-line spacing (115 MHz) —appears to be adequate for resolving the CS_2_ Doppler broadened absorption lines.

**Figure 4:**
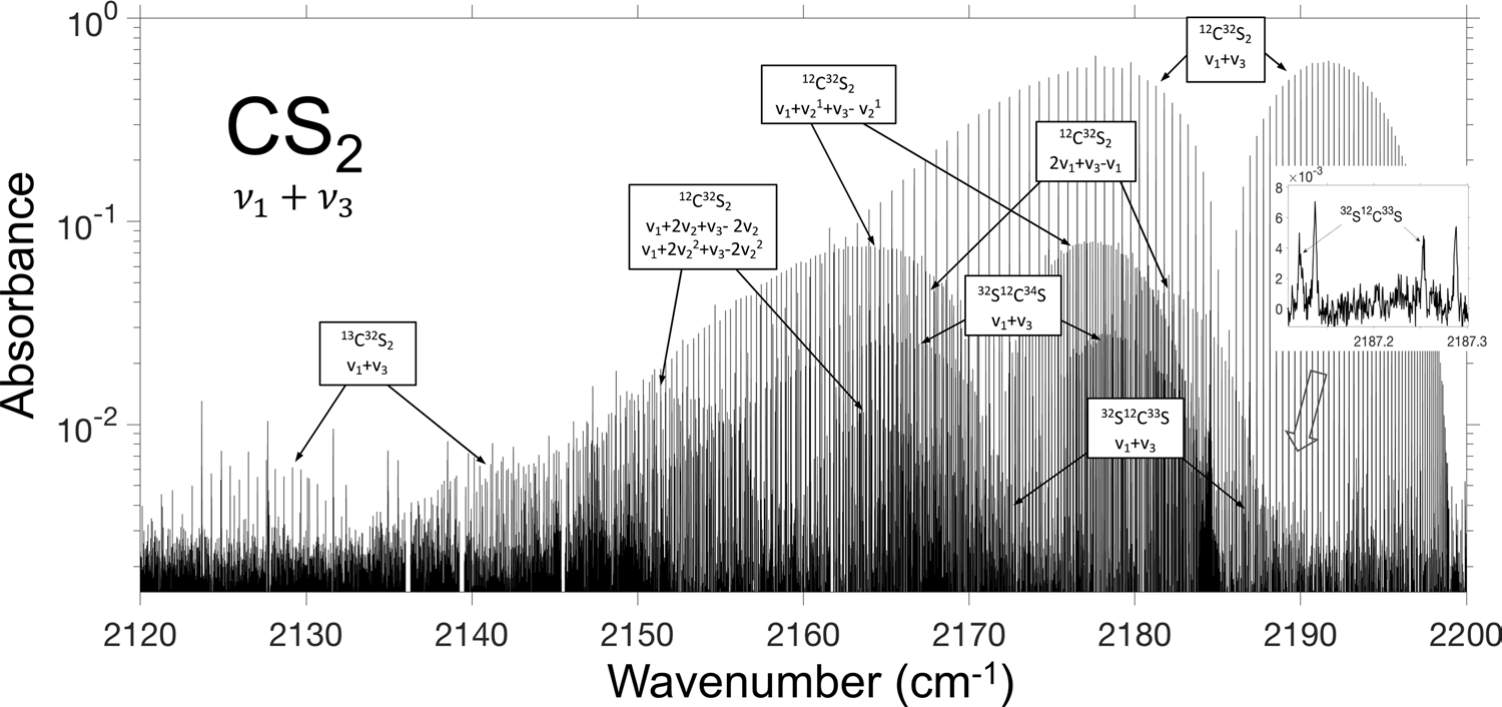
Log-scale spectrum corresponding to the ν_1_ + ν_3_ band of CS_2_ that include: bands due to isotopes: ^34^S, ^33^S, and ^13^C and hot bands from the thermally excited vibrational states ν_1_ and ν_2_. The inset shows two peaks due to the low-abundance ^33^S isotope. For better visibility of the spectrum, the CO and N_2_O lines were digitally subtracted via HITRAN-based simulations. Also, few slices between 2135 and 2165 cm^-1^ corresponding to strong water absorption outside the gas cell were manually cut out.

Figure 4 shows the absorbance spectrum of CS_2_ plotted on a log-scale that allows to see the weaker bands. For better visibility of the CS_2_ spectrum, the lines of CO and N_2_O present in the mixture due to the room air were digitally subtracted via HITRAN simulations using concentration as the only fitting parameter. This was also used to verify the absolute accuracy of our frequency readings: the positions of the CO and N_2_O peaks deviated from those obtained from HITRAN by less than 1 MHz and allowed clean removal of these peaks. Additionally, few slices between 2135 and 2165 cm^−1^—corresponding to strong peaks of water vapor outside the gas cell—were manually cut out in Fig. 4.

The high resolution and long coherent averaging allowed us to identify 981 spectral lines of the ν_1_ + ν_3_ absorption band of CS_2_. In fact, because of the low noise of the baseline, we were able to detect CS_2_ lines that were about 10^3^ times weaker than the strongest ones (see the inset to Fig. 4). In addition to the main molecule, we were able to resolve the spectra of three isotopologues (^34^S^12^C^32^S, ^33^S^12^C^32^S, and ^32^S^13^C^32^S), as well as the spectra of seven hot bands due to transitions from the thermally excited vibrational states, labelled in Fig. 4. The inset shows the two peaks corresponding to the low-abundance ^33^S isotope (~ 0.8% with respect to ^32^S), along with the two peaks for the main molecule. More details on our CS_2_ measurements, including line lists and the interpretation of the bands can be found in^43^.

### OCS molecule

**Figure 5:**
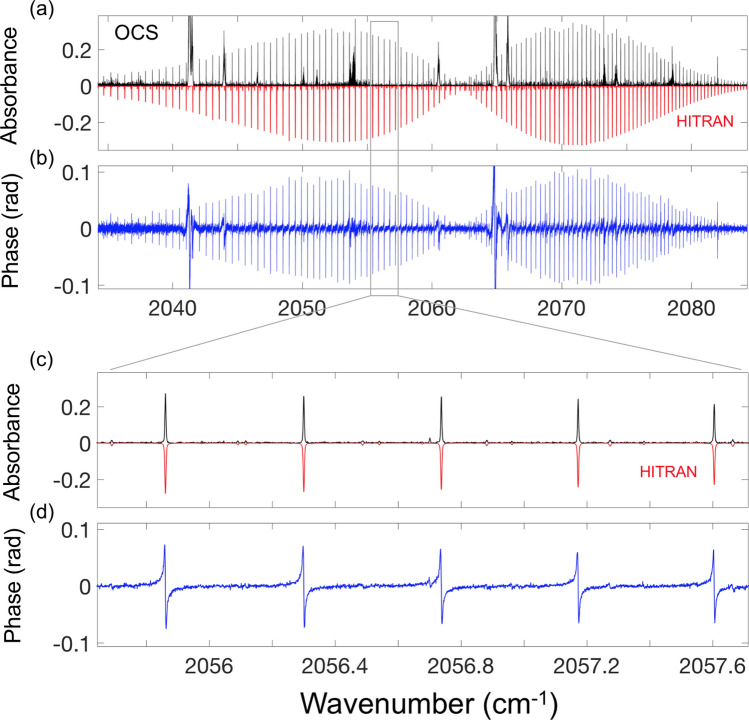
(**a**) Absorbance and (**b**) phase spectrum for the ν_3_ asymmetric band of OCS. The noise features (e.g. at 2041, 2065 cm^−1^ etc.) are related to strong (and broad) water absorption lines outside the gas cell. (**c**,**d**) Expanded OCS spectrum for the absorbance and phase. Shown in (**a**,**c**) are simulated HITRAN-based absorbance spectra, inverted for clarity.

Figure 5 presents the portion of the spectrum (absorbance and phase) from the same experiment that reveals the presence of trace amounts of carbonyl sulfide (OCS) molecule, although this molecule was not originally present in the gas mixture. The plot also shows the simulated (HITRAN) spectrum for the asymmetric ν_3_ stretch of OCS. From the known absorption cross section of OCS, its concertation was evaluated to be 1.75 ppm. Our repeated measurements revealed that the OCS concertation kept rising at a rate of approximately 15% per day, while the concertation of CS_2_ was decreasing at a rate of 0.5–0.7% per day. We hypothesize that the emergence of OCS is due to a chemical oxidation reaction of CS_2_ in the presence of O_2_ in the gas cell^44^.

## Discussion

Using a highly-coherent and broadband dual-comb spectroscopy system based on subharmonic generation, we demonstrate the acquisition of up to 2.5 million spectral elements over the wavelength range of 3.17–5.13 µm (frequency span 1200 cm^-1^)—by interleaving spectra with discretely stepped comb-line spacing. Among other molecular absorption features, we fully resolved the Doppler-broadened spectra corresponding to the strongest 981 transitions of the ν_1_ + ν_3_ band (4.5–4.7 µm) of CS_2_ and its three isotopologues with ~ 50-kHz accuracy of the absolute frequency referencing. Most of the line intensities were accurately measured for the first time and will be included in the line lists for molecular databases^43^. Furthermore, the obtained data can be used as a testing ground for theoretical models. The sampling point spacing in our experiment can be further reduced (at the expense of the measurement time) by 2–3 orders of magnitude, limited only by the 50-kHz uncertainty in the absolute frequency. This in turn can be further improved using a more stable narrow-linewidth optical reference laser and a tighter feedback loop.

## Methods

### OPO comb modes and RF-to-optical frequency mapping

**Figure 6:**
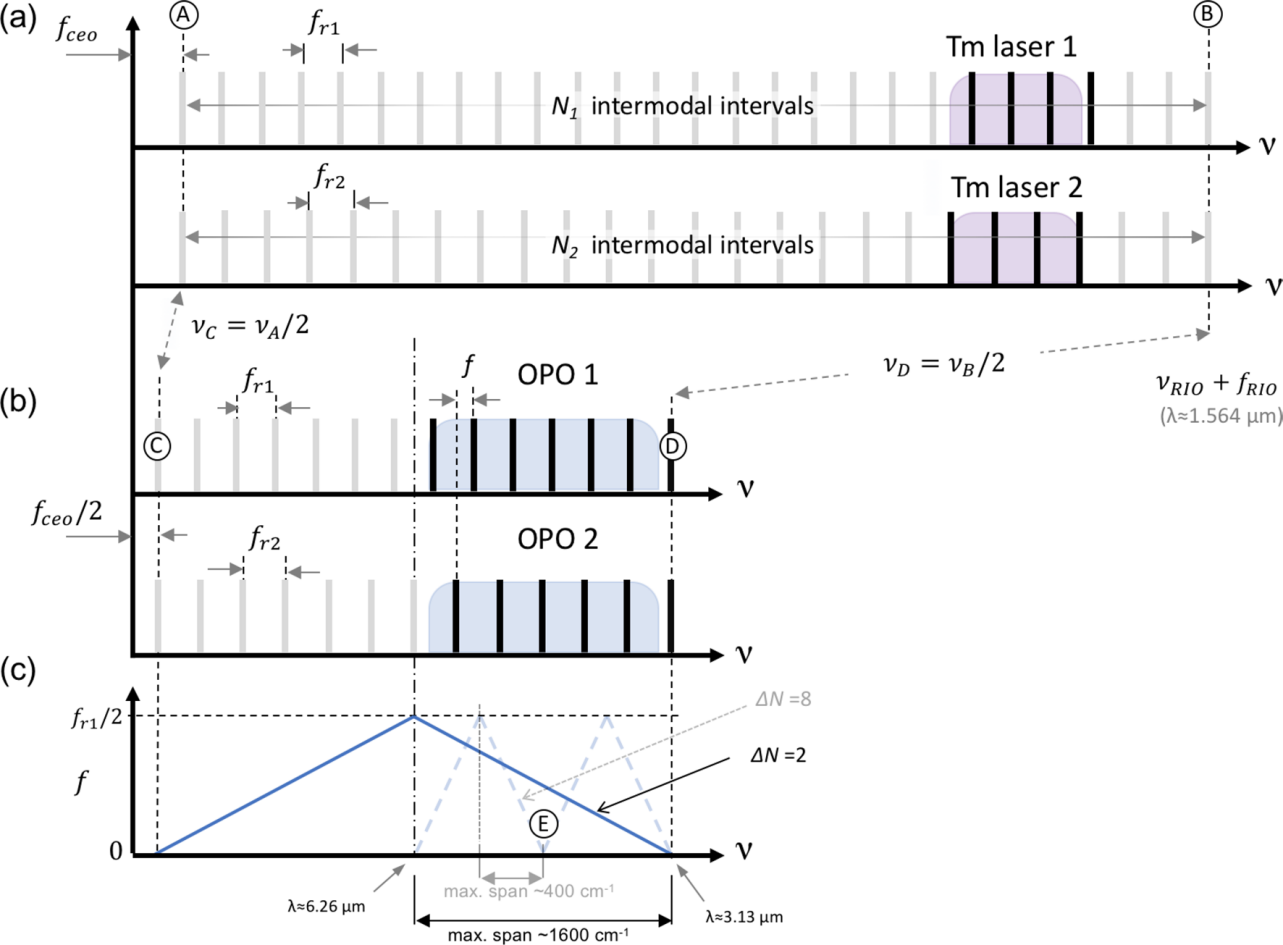
(**a**) Comb lines extrapolated to zero optical frequency ν for the two Tm-fiber pump lasers, with the repetition rates *f*_r1_ and *f*_r2_ respectively. The Tm combs are $f_{ceo}$ stabilized and phase locked to a common narrow-band ‘RIO’ laser (λ≈1.564 µm) so that their teeth overlap at the two lock points ‘A’ and ‘B’. (**b**) The subharmonic OPO combs are phase locked to the pump and their teeth (e.g. if Δ*Ν* = 2 and *N*_1,2_ are even) overlap at the anchor point ‘C’ distanced by *f*_ceo_/2 from zero, and at a common anchor point ‘D’ (λ≈3.13 µm). (**c**) RF-to-optical frequency mapping for Δ*Ν* = 2 and Δ*Ν* = 8.

The comb modes for both Tm-fiber pump lasers were locked, at the two common anchor points (Fig. 6): near zero frequency with a common carrier-envelope offset (CEO) $f_{ceo}^{pump}$ =  + 190 MHz (point A), and near the frequency of a narrow-linewidth (~ 3 kHz) continuous wave (CW) reference ‘RIO’ semiconductor laser at near λ≈1564 nm with an offset $f_{RIO}$ =  + 140 MHz (point B). Between A and B, there are *N*_1_ intermodal intervals (*f*_r1_) for laser 1 and *N*_2_ intervals (*f*_r2_ > *f*_r1_) for laser 2, such that *N*_1_ × *f*_r1_ = *N*_2_ × *f*r_2_ and

1 $${\Delta }f_{r} = f_{r2} - f_{r1} = \frac{{N_{1} - N_{2} }}{{N_{2} }}f_{r1} = \frac{{{\Delta N}}}{{N_{2} }}f_{r1} = \frac{{{\Delta N}}}{{N_{1} }}f_{r2} .$$

The repetition rate offset ${\Delta }f_{r}$ between the two lasers is quantized. For example, for Δ*N* = 2, ∆*f*_r_ ≈ 138.5 Hz and for even *N*_1,2_ such that *N*_1_ = 2(*k*_0_ + 1) and *N*_2_ = 2*k*_0_ with *k*_0_ an integer, the ratios *f*_r1_/Δ*f*_r_ and *f*_r2_/Δ*f*_r_ are integer numbers. In fact, in our experiments the measured ratio *f*_r1_/Δ*f*_rep_ (≈*k*_0_) was an integer: 831,844 with a remainder after division < 10^–3^.

Both OPO combs were running in the frequency-divide-by-2 mode with respect to the pump, such that that CEO frequency for both of them (anchor point C in Fig. 6 was half of that of the pump CEO:

2 $$f_{ceo}^{OPO1} = f_{ceo}^{OPO2} = \frac{1}{2}f_{ceo}^{pump} .$$

Another anchor point (D) is at ν_D_ = ½ ν_B_ (near ~ 3.13 µm), just above (in frequency) our broadest spectral span. For Δ*N* = 2 we used the following RF-to-optical mapping for the sensing comb:

3 $$\nu = \nu_{D} - \frac{{f_{r2} }}{{\Delta f_{r} }}f = \frac{1}{2}f_{ceo}^{pump} + \frac{{f_{r2} }}{{\Delta f_{r} }}\left( {f_{r1} - f} \right),$$

where ν is the optical frequency that interrogates the sample, and *f* is RF frequency. The whole 1200 cm^-1^ spectral span is thus mapped to the 1–48-MHz RF range. In a similar way, with the reduced (2030–2300 cm^-1^) frequency coverage, selected for the study of CS_2_ molecule, we have used Δ*N* = 8, ${\Delta }f_{r}$ = 554 Hz, such that the RF comb’s span matches the aliasing free bandwidth, with the following RF-to-optical frequency mapping for the sensing comb:

4 $$\nu = \frac{1}{2}f_{ceo}^{pump} + \frac{{f_{r2} }}{{\Delta f_{r} }}\left( {3f_{r1} - f} \right).$$

Here, the term $\frac{1}{2}f_{ceo}^{pump} + 3\frac{{f_{r1} f_{r2} }}{{{\Delta }f_{r} }}$ corresponds to the anchor point “E” in Fig. 6c for both OPO combs, and $\frac{{f_{r2} }}{{{\Delta }f_{r} }}$ is the RF-to-optical upscaling factor (with a negative sign).

Remarkably, with the known Δ*N*, frequency offsets $f_{ceo}^{pump} { }$ and $f_{RIO}$, and with just two readings from the frequency counters ($f_{r1}$ and ${\Delta }f_{r}$), we were able to determine the vacuum wavelength of the RIO laser with the fractional accuracy of better than 10^–9^.

### Stabilization and stepping of the RIO laser and the absolute frequency referencing

Since the two pump Tm-fiber combs are phase locked to a common optical reference (RIO laser), and the OPO combs, in turn, are phase-locked to the Tm combs, the drifts of the RIO laser frequency ($\nu_{RIO} )$, typically ~ 10 MHz during a day, affect the absolute position of the comb lines. To avoid these drifts, we implemented an active $\nu_{RIO}$ stabilization. Using the readings of a frequency counter referenced to a Rb clock for the repetition rate ($f_{r1}$) of one of the Tm-fiber combs, and applying the difference between the measured and a desired repetition rate as a feedback to act on the RIO laser temperature with a slow servo loop with an update time of 4 s, we achieved the standard deviation for $f_{r1}$ of 0.09 Hz (${\upsigma }f_{r1} /f_{r1} { } = 7.8 \times 10^{ - 10}$), which corresponds to the standard deviation of the RIO laser frequency ${\upsigma }\nu_{RIO} = \nu_{RIO} \times {\upsigma }f_{r1} /f_{r1} \approx$ 150 kHz. Since both OPO combs are pinned to a common frequency offset near zero, the frequency uncertainty of a comb line is proportional to its absolute frequency. For example, for the CS_2_ absorption band near 4.6-µm, the standard deviation is $\sigma \nu = \nu \times 7.8 \times 10^{ - 10} \approx$ 50 kHz.

The interleaved DCS spectra were acquired by stepping, through temperature control, $\nu_{RIO}$ in eight 42.35-MHz increments (corresponding to $f_{r1}$ steps of ≈25 Hz), resulting in the comb-line shifts that vary from 21 MHz (at ≈3.2 µm) to 13 MHz (at ≈ 5.1 µm), and were 14.4 MHz (about 1/8 of the original 115-MHz comb-line spacing) near the 4.6-µm absorption band of CS2.

### CS_2_ sample preparation

A droplet of liquid-phase CS_2_ dissolved in n-hexane at a concentration of 100.6 μg/ml ± 2% (Chem Services, Inc.) was placed in a 10-ml-volume chemical flask and fully evaporated, together with the ambient room air in the headspace, into the evacuated 0.5-L multipass gas cell. The resulting pressure in the gas cell was then measured with an accuracy of ± 1%. Next, the same procedure was repeated with the chemical flask filled just with room air. The fractional (by molecules) concentration of the hexane-CS_2_ mixture in the gas cell was determined by comparing the measured pressure in the gas cell due to the mixture (liquid droplet plus air) and the pressure produced due to the flask filled just with air. (In fact, there were comparable concentrations of molecules coming from the droplet and room air.) For spectral measurements, the gas cell was further evacuated to get the total pressure of 6.03 mbar, with the calculated CS_2_ concentration of 94.6 ppm ± 3% (part-per-million by volume). The gas cell temperature was kept at 293.7 ± 0.5 K.
